# Association of Delta Neutrophil Index with the 30-day Mortality in Adult Cardiac Surgical Patients

**DOI:** 10.7150/ijms.97400

**Published:** 2024-07-01

**Authors:** Sujin Kim, Ji-Hyoung Park, Hyunjae Lim, Haesung Lee, Seung Woo Song

**Affiliations:** 1 Wonju Severance Christian Hospital, Republic of Korea.; 2 Wonju College of Medicine, Yonsei University, Republic of Korea.

**Keywords:** delta neutrophil index, cardiac surgical procedures, perioperative care, critical care, blood cell count

## Abstract

**Purpose:** This study aimed to assess the predictive accuracy of 30-day mortality with delta neutrophil index (DNI) in adult cardiac surgical patients.

**Methods:** This study enrolled patients who underwent cardiac surgery under general anesthesia between March 2016 and May 2022 at a tertiary hospital in the Republic of Korea. DNI was measured preoperatively, on postoperative arrival to the surgical intensive care unit (ICU), and 12, 24, 48, and 72 h postoperatively. Receiver operating characteristic (ROC) analysis was employed to identify the prediction accuracy of DNI. An area under ROC curve (AUROC) ≥0.700 was defined as satisfactory predictive accuracy. An optimal cutoff point for the DNI value to maximize predictive accuracy was revealed in the ROC curve, where [sensitivity + specificity] was maximum.

**Results:** This study included a total of 843 patients in the final analyses. The mean age of the study population was 66.9±12.2 years and 38.4% of them were female patients. The overall 30-day mortality rate was 5.2%. Surgery involving the thoracic aorta, history of prior cardiac surgery, or emergency surgery were associated with a higher mortality rate. The DNI showed satisfactory predictive accuracy at 24 h, 48 h, and 72 h postoperatively, with AUROC of 0.729, 0.711, and 0.755, respectively. The optimal cutoff points of DNI at each time point were 3.2, 3.8, and 2.3, respectively.

**Conclusions:** Postoperative DNI is a good predictor of 30-day mortality after cardiac surgery and has the benefit of no additional financial costs or time.

## Introduction

Patients who undergo cardiovascular surgery frequently experience postoperative complications due to preoperative comorbidities and the high-risk nature of cardiovascular surgeries 1. The incidence of postoperative mortality remains higher than that of other surgeries. Mortality following valve replacement and coronary artery bypass was reported to be 2-3% and 4%, respectively 2. Identifying patients at higher risk of complications during the early postoperative phase from lower-risk patients, followed by appropriate treatment, can improve survival.

The delta neutrophil index (DNI) is the fraction of immature granulocytes in circulating blood obtained from the differences in subfractions measured in the myeloperoxidase and nuclear lobularity channels 3. It has been widely studied for predicting the clinical course of high-risk patients who experience a systemic inflammatory response or those undergoing major noncardiac surgeries 4-6. However, only a few studies have assessed the use of DNI in patients undergoing cardiac surgery. Hence, this study examined the role of the DNI in predicting postoperative mortality among patients undergoing cardiac surgery.

## Materials and Methods

### Study population

This study enrolled patients who underwent cardiovascular surgery under general anesthesia between March 2016 and May 2022 at a tertiary university hospital in Wonju, Republic of Korea. Inclusion criteria were as follows: cardiac valve replacement, cardiac valvuloplasty, coronary artery bypass grafting (CABG), other cardiac surgical procedures requiring cardiopulmonary bypass (CPB), and surgery involving the thoracic aorta. Exclusion criteria were as follows: preoperative DNI > 7.0%, 5 DNI never measured during the first three postoperative days, intraoperative death, and cardiac surgeries without CPB, except in cases of off-pump CABG (OPCAB). The patients were routinely administered prophylactic antibiotics preoperatively.

### Variables

The independent variable used was the DNI values measured until the postoperative day (POD) 3. The DNI was automatically measured as (the leukocyte subfraction assayed in the myeloperoxidase channel on cytochemical reaction) - (the leukocyte subfraction assayed in the nuclear lobularity channel using the reflected light beam) by a hematological analyzer, ADVIA 2120i (Siemens Healthcare Diagnostics, Inc., New York, USA). It was reported as one item of complete blood cell count (CBC) result, and CBC was conducted 2 hours before surgery, on postoperative arrival to the surgical intensive care unit (ICU), and 12, 24, 48, and 72 hours postoperatively. The primary outcome was the 30-day mortality. Age, sex, height, weight, history of prior cardiac surgery, diabetes, and preoperative hemoglobin, creatinine, and BNP (Brain natriuretic peptide) levels were recorded as covariates. The duration of CPB or aortic cross-clamp (ACC), wherein both were performed, and anesthesia duration were also recorded. Neutrophil to lymphocyte ratio (NLR) and neutrophil to lymphocyte*platelet ratio (NLPR) in each time point was calculated based on the CBC result 7.

### Statistical analysis

Descriptive analyses were performed according to patient survival or mortality. The t-test or Wilcoxon rank-sum test was conducted according to the distribution of each continuous variable. The chi-squared test was used for comparing categorical variables.

Receiver operating characteristic (ROC) curve analysis was performed to identify the prediction accuracy of DNI for the primary outcome. An AUROC of 0.700 or higher was defined as satisfactory predictive accuracy. An optimal cutoff point for this value to maximize predictive accuracy was revealed in the ROC curve, where [sensitivity + specificity] was maximum. R statistics version 4.3.1 (R Core Team, Vienna, Austria) was used for statistical analysis and graphical presentation.

Multivariate logistic regression analysis was conducted to determine an association of DNI with the 30-day mortality considering the effect of covariates. Covariates were emergency operation, history of prior cardiac surgery, anemia of Hb < 10g/dL, serum creatinine > 1.3mg/dL, BNP > 100pg/mL, and CPB duration. The analysis was conducted by enter method and backward variable elimination.

## Results

**Figure 1:**
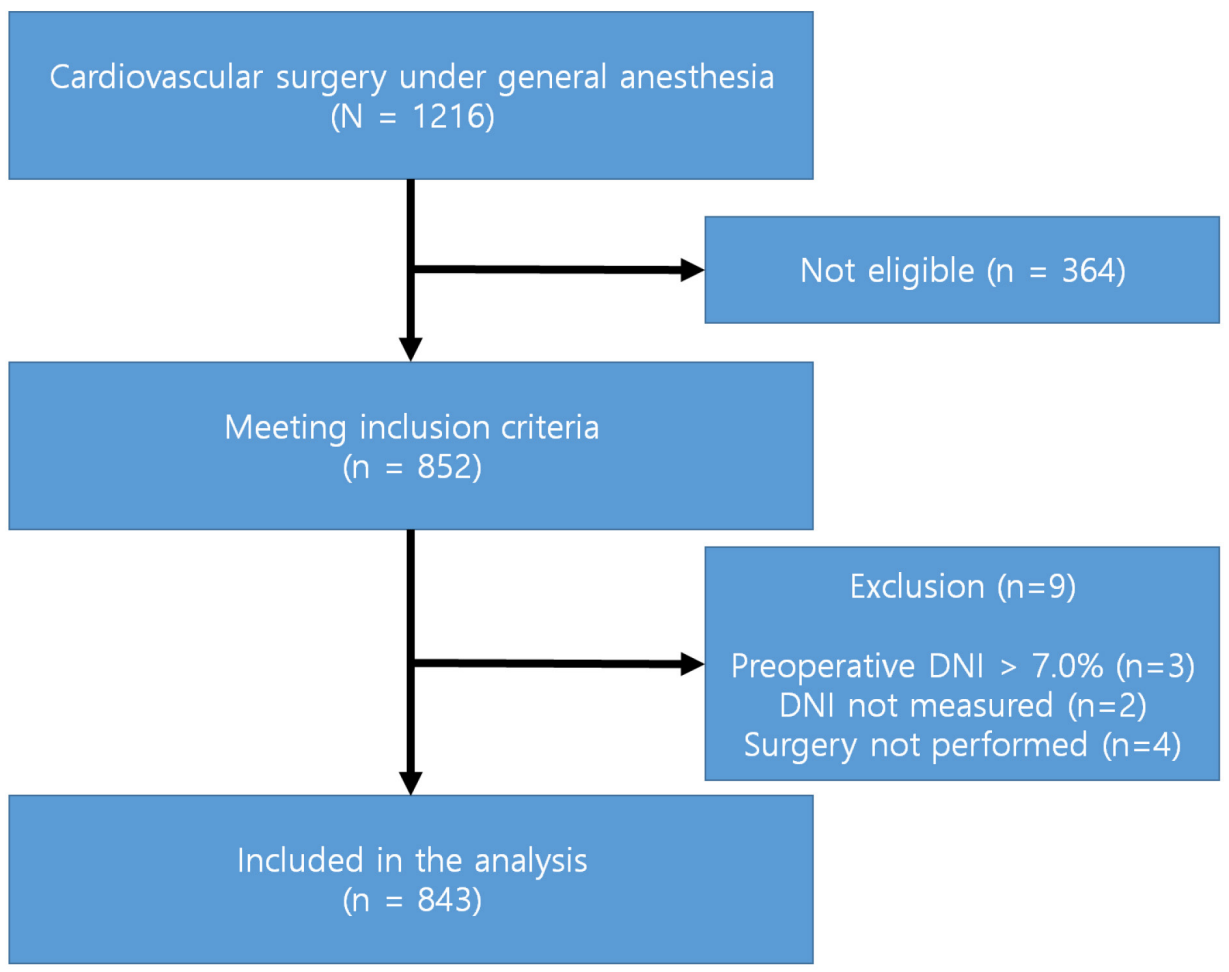
Study flow diagram.

A total of 852 patients met the inclusion criteria. Nine patients were excluded according to the exclusion criteria. The preoperative DNI of the three surgical cases was >7.0%. The postoperative DNI was not measured in two cases. Surgical procedures were not performed after inducing anesthesia in four cases. Hence, a total of 843 patients were included in the final analysis (Figure 1).

In this population, the overall 30-day mortality rate was 5.2%. The mean age of the whole patients was 66.9 ± 12.2, wherein 38.4% are female patients. The mean body weight was 62.1 ± 11.7 kg, and the body mass index was 24.1 ± 3.5 kg/m^2^ (Table 1). Anthropometric and demographic data were similar between survivors and deceased individuals, except for age.

Off-pump coronary artery bypass (OPCAB) was the most frequently performed procedure in the medical center, and 9 patients underwent unplanned cardiopulmonary bypass. Only four cases of CABG with CPB were done in the initial plan. Mortality in 30 days postoperatively was less frequent in patients who underwent OPCAB (2.1%) compared with the entire population.

Valve surgery was also frequently performed, with similar frequencies in both groups. OPCAB was more frequent in the survival group, while surgical procedures involving the thoracic aorta were more frequent in the mortality group. Mortality in 30 days postoperatively was 15.0% among patients who underwent surgery involving the thoracic aorta. Emergency cardiac surgery was associated with a higher mortality rate than elective surgery (17.2% vs. 3.7%). Patients with prior experience of cardiac surgery comprised 5.0% of the population, with a 19.0% mortality rate.

Preoperative features differed between the survival and mortality groups in terms of age, presence of prior cardiac surgical history, and hemoglobin, creatinine, and BNP levels. ACC and CPB durations were longer in the mortality group.

**Figure 2:**
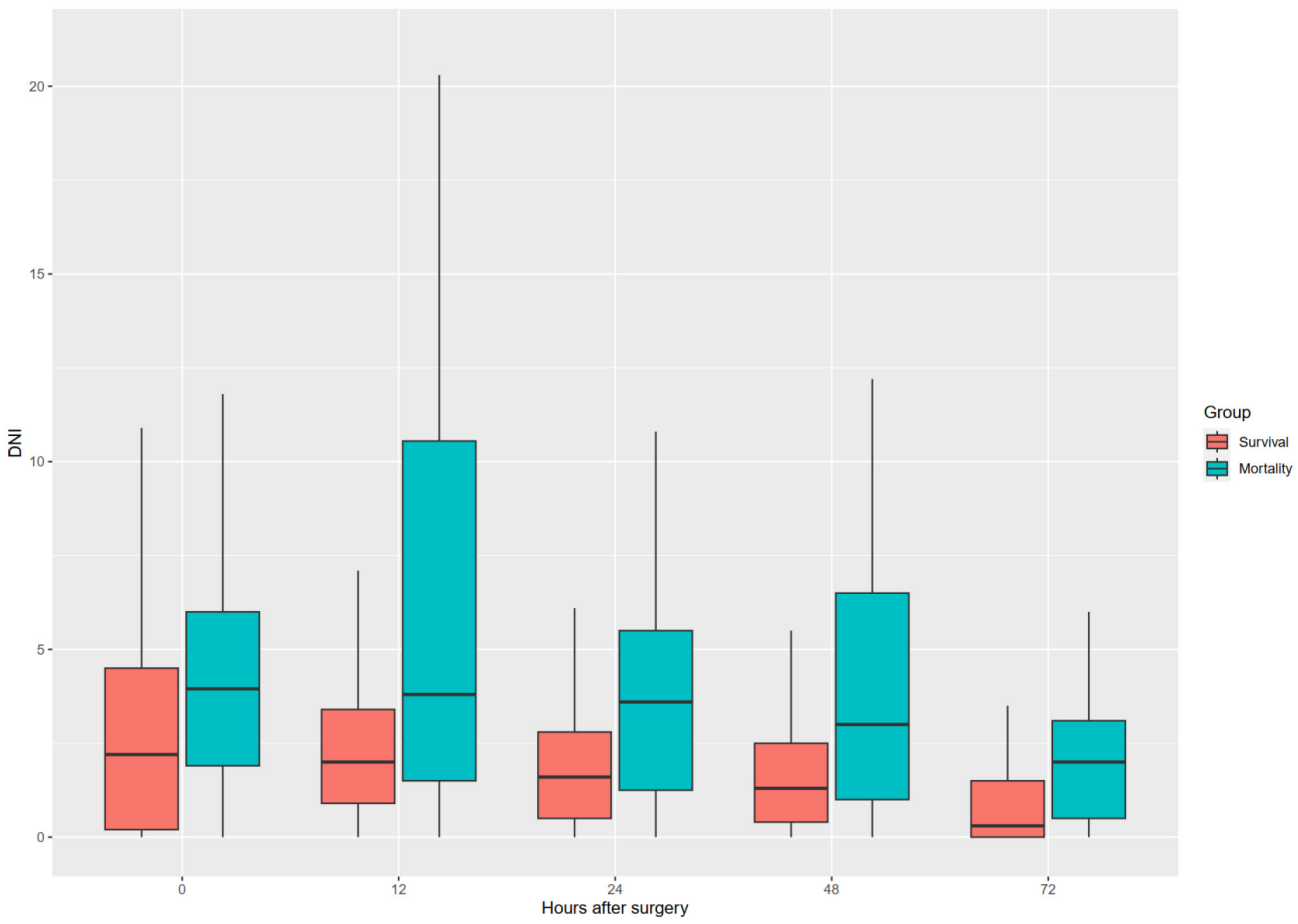
DNI trend through a postoperative period in both groups.

There was a uniform trend of higher DNI in the mortality group from the immediate postoperative period until POD 3 (Figure 2). Platelet count was the only hematologic factor that was uniformly lower in the mortality group compared with the survival group (Table 2). The neutrophil to lymphocyte*platelet ratio was higher at 24 h postoperatively; however, it was lower in the mortality group compared with the survival group during the immediate postoperative period.

**Figure 3:**
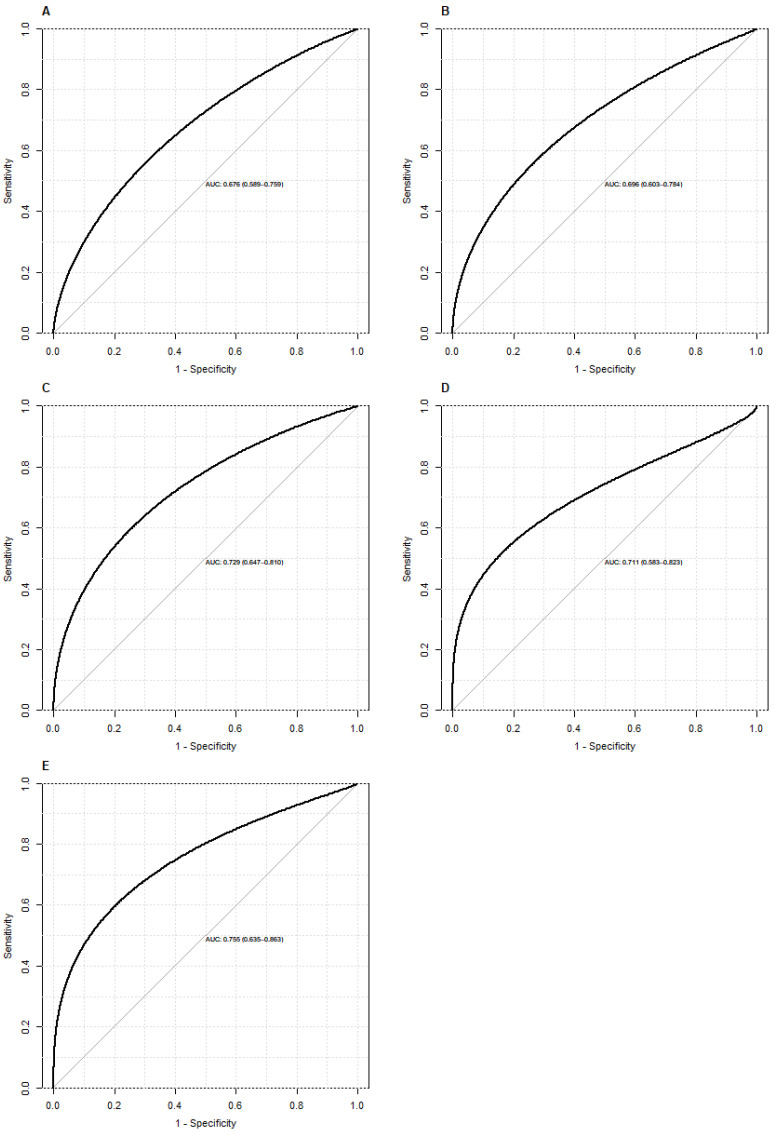
A ROC curve of DNI for postoperative mortality at each time point. **A,** On admission to the ICU; **B,** 12 h postoperatively; **C,** 24 h postoperatively; **D,** 48 h postoperatively; **E,** 72 h postoperatively.

On the ROC curve analysis, DNI showed fair prediction accuracy of 30-day mortality at 24 h, 48 h, and 72 h postoperatively (Figure 3). Specificity was higher than 80% from 12 h to 72 h postoperatively (Table 3).

Multivariate logistic regression analysis revealed statistically significant association of DNI, postoperative 12 hours to 72 hours, and the 30-day mortality (Table 4 and Supplementary material 1). Emergency operations and longer CPB duration were also significantly associated with a higher risk of mortality.

## Discussion

Overall, the DNI showed fair performance in discriminating the mortality group from the survival group among patients who underwent cardiac surgery. DNI and platelet counts indicated consistent differences at each time point. However, the platelet count can be altered through transfusion or hemodilution 8. The DNI is not altered by these interventions and can be adopted as a reliable prognostic marker in patients undergoing major surgeries 4, 9.

In a study by Kong et al., the DNI was associated with 30-day mortality among patients with ST-segment elevation myocardial infarction 10. A DNI > 2.9% at 24 h after admission could predict 30-day mortality in patients. This DNI threshold value is similar to the value proposed in the current study of the equivalent time point, 3.2%.

During cardiac surgery, inflammation is induced by various factors such as surgical trauma, ischemia, and CPB 11. Platelets are activated by heparin, hypothermia, and CPB, which amplify tissue injury. Cardiac surgery is linked with a systemic inflammatory response that can lead to adverse clinical outcomes, and the incidence of clinically defined SIRS was 96.2% in the first 24 h after admission in a previous study 12. This may be related to the good performance of the DNI as a predictive marker, considering that DNI predicts systemic inflammation outcomes 13.

The overall characteristics of the surgical population were similar to those reported in previous studies. Surgery involving thoracic aorta, and emergency cardiac surgery were associated with higher mortality 14, 15. Re-do cardiac surgery is another high-risk factor 16. A higher NLR was also associated with poor outcome, which was formerly reported by the previous studies 7, 17.

CBC is routinely measured in cardiac surgical patients postoperatively 18, and DNI is easily automatically measured during CBC testing using a blood cell analyzer 19. The decisions of clinicians who have the burden of postoperative care in cardiac surgical patients can be assisted by the DNI without additional cost and time.

One limitation of our study is its retrospective nature, necessitating validation through prospective research. A multicenter study is warranted to identify its usefulness in a cardiac surgery population with other preoperative demographics or medical features.

## Supplementary Material

Supplementary table.

## Figures and Tables

**Table 1:** Baseline information based on 30-day survival or mortality

	Survival group (n = 799)	Mortality group (n = 44)
Age, years **	66.6±12.1	72.0±11.4
**Female**	305 (38.2)	19 (43.2)
**Body weight, kg**	62.1±11.7	62.1±11.0
**Body mass index, kg/m^2^**	24.0±3.5	24.5±3.9
Emergency surgery***	77 (9.6)	16 (36.4)
History of cardiac surgery***	34 (4.3)	8 (18.2)
**Diabetes**	299 (37.4)	14 (31.8)
**Diabetes with insulin therapy**	55 (6.9)	3 (6.8)
Preoperative Hemoglobin, g/dL ***	12.1±1.9	10.8±2.1
Preoperative serum creatinine, g/dL ***	0.93 (0.77-1.15)	1.16 (0.91-2.03)
Preoperative BNP***	159.1 (53.13-488.3)	345.71 (125.8-1238.1)
**Duration of procedure, min**		
Anesthesia ***	330 (290-380)	387.5 (313.8-502.5)
CPB ***	125 (91.8-176.3)	230 (111-297.5)
ACC **	84.5 (55-120.3)	128 (67-191)
**Fluid intake per anesthesia time, mL/min**	6.6 (5.0-8.2)	6.1 (5.3-7.9)
Total fluid intake, mL**	2150 (1600-2800)	2575 (2012-3300)
Transfusion of packed red blood cells, unit***	1 (0-2)	3.5 (2-4.3)
**Surgery**		
OPCAB**	320 (40.1)	7 (15.9)
**Isolated single-valve procedure**	264 (33.0)	13 (29.5)
Surgical involvement of thoracic aorta***	34 (4.3)	6 (13.6)

Data were presented as mean±SD, median (IQR), or frequency (percent). *p < 0.05, **p < 0.01, ***p < 0.001. BNP, Brain natriuretic peptide; CPB, cardiopulmonary bypass; ACC, Aortic cross-clamp; OPCAB, off-pump coronary bypass grafting

**Table 2:** Postoperative hematologic study results based on 30-day survival or mortality

	Survival group (n = 799)	Mortality group (n = 44)	W	*P* value
**On admission to the ICU**				
WBC, per μL	9430 (6305-12800)	8675 (4423-10795)	20226	NS
Neutrophils, per μL	7780 (5195-10770)	7550 (3420-10370)	19027	NS
Lymphocytes, per μL*	830 (510-1395)	580 (480-965)	21363	0.007
Platelets, per nL***	146 (115-188.5)	110 (84.75-132.75)	24920	< 0.0002
NLR	9.39 (6.78-15.45)	11.60 (8.98-18.63)	14452	NS
NLPR***	10.28 (6.29-18.96)	5.63 (3.29-10.42)	11077	< 0.0002
DNI***	2.2 (0.2-4.6)	4.1 (2.1-11.0)	11114	< 0.0002
**Postoperative, 12 hrs**				
WBC, per μL	11760 (9360-14900)	10130 (8332-13387)	19827	NS
Neutrophils, per μL	10480 (8170-13430)	9130 (7410-11740)	19438	NS
Lymphocytes, per μL	550 (390-770)	580 (430-820)	15652	NS
Platelets, per nL***	146 (113-189)	104 (51.25-134.25)	25081	< 0.0002
NLR	20.57 (13.76-33.59)	18.00 (11.68-29.59)	18302	NS
NLPR	12.81 (7.15-23.48)	17.30 (10.78-26.46)	13313	NS
DNI***	2.0 (0.9-3.5)	4.8 (1.7-11.2)	10250	< 0.0002
**Postoperative, 24 hrs**				
WBC, per μL	12200 (9565-15165)	11660 (8650-15920)	16032	NS
Neutrophils, per μL	10415 (7995-13275)	10140 (7270-14240)	15602	NS
Lymphocytes, per μL *	800 (580-103)	630 (510-800)	18611	0.0073
Platelets, per nL ***	131 (99.5-174.5)	100 (70-119)	20978	< 0.0002
NLR	15.23 (10.40-22.11)	17.48 (11.07-27.37)	12762	NS
NLPR **	10.18 (5.52-19.54)	16.95 (10.74-29.55)	9678	0.0004
DNI ***	1.6 (0.5-2.9)	4.0 (1.4-10.7)	8212.5	< 0.0002
**Postoperative, 48 hrs**				
WBC, per μL	12210 (9830-15330)	12850 (8688-15595)	11130	NS
Neutrophils, per μL	10290 (8010-13060)	11240 (7997-13555)	10550	NS
Lymphocytes, per μL***	960 (690-1280)	605 (455-872)	16402	< 0.0002
Platelets, per nL**	118 (83.5-167)	73.5 (56.5-114.2)	15740	0.0002
NLR***	12.34 (9.14-17.80)	19.29 (15.24-24.81)	6347	< 0.0002
NLPR***	9.26 (5.22-17.70)	19.13 (11.56-38.41)	5604	< 0.0002
DNI**	1.3 (0.4-2.5)	3.5 (1.0-9.1)	6766	0.0004
**Postoperative, 72 hrs**				
WBC, per μL	9950 (8065-12285)	11320 (10440-14790)	7971.5	NS
Neutrophils, per μL*	7920 (6080-10050)	9710 (8432-13012)	7140.5	0.0069
Lymphocytes, per μL***	1030 (740-1400)	690 (490-935)	15251	< 0.0002
Platelets, per nL***	120 (84-170)	63.5 (39.2-108.7)	15230	< 0.0002
NLR***	9.38 (6.93-13.57)	16.44 (13.21-23.86)	3985	< 0.0002
NLPR***	6.32 (3.61-12.56)	21.48 (10.11-54.12)	3560	< 0.0002
DNI***	0.3 (0.0-1.5)	2.5 (0.7-4.5)	5058.5	< 0.0002

Bonferroni correction was performed. *p < 0.01, **p < 0.002, ***p < 0.0002. NS, Not significant; ICU, intensive care unit; WBC, white blood cell; NLR, neutrophil to lymphocyte ratio; NLPR, neutrophil to lymphocyte*platelet ratio; DNI, delta neutrophil index.

**Table 3:** Performance of postoperative DNI as a predictor of 30-day mortality

Operating point	Value
**On admission to the ICU**	
AUROC (95% CI)	0.676 (0.591-0.761)
DNI threshold value	3.4
Sensitivity / Specificity, %	67.4 / 65.5
PPV / NPV, %	2.6 / 90.5
**At postoperative 12 hrs**	
AUROC	0.700 (0.607-0.779)
DNI threshold value	4.7
Sensitivity / Specificity, %	51.2 / 84.3
PPV / NPV, %	2.9 / 85.6
**At postoperative 24hrs**	
AUROC	0.730 (0.641-0.814)
DNI threshold value	3.2
Sensitivity / Specificity, %	62.2 / 78.9
PPV / NPV, %	2.2 / 88.0
**At postoperative 48 hrs**	
AUROC	0.711 (0.586-0.829)
DNI threshold value	3.8
Sensitivity / Specificity, %	50.0 / 90.3
PPV / NPV, %	1.9 / 84.6
**At postoperative 72 hrs**	
AUROC	0.755 (0.639-0.866)
DNI threshold value	2.3
Sensitivity / Specificity, %	53.8 / 89.2
PPV / NPV, %	1.7 / 86.0

**Table 4:** Result of logistic regression analysis by backward variable elimination

Variable	Coefficient	95% CI of coefficient	P value
**On admission to the ICU**			
DNI, %	1.06	1.00-1.12	NS
Emergency surgery***	5.66	2.36-13.58	< 0.0002
Hb < 10g/dL*	3.57	1.55-8.23	0.0028
CPB duration, minute***	1.01	1.01-1.02	< 0.0002
**Postoperative, 12 hrs**			
DNI, %***	1.11	1.05-1.17	< 0.0002
Emergency surgery**	5.13	2.04-12.9	0.0005
Hb < 10g/dL*	3.60	1.53-8.49	0.0034
CPB duration, minute***	1.01	1.01-1.02	< 0.0002
**Postoperative, 24 hrs**			
DNI, %**	1.10	1.04-1.16	0.0006
Emergency surgery**	4.63	1.78-12.02	0.0016
Hb < 10g/dL*	3.30	1.37-7.98	0.0079
CPB duration, minute***	1.01	1.00-1.01	< 0.0002
**Postoperative, 48 hrs**			
DNI, %***	1.18	1.08-1.28	< 0.0002
Emergency surgery**	5.59	1.88-16.57	0.0019
Hb < 10g/dL	3.13	1.10-8.89	NS
CPB duration, minute	1.01	1.00-1.01	NS
**Postoperative, 72 hrs**			
DNI, %***	1.45	1.19-1.76	< 0.0002
Emergency surgery**	6.18	2.05-18.65	0.0012
CPB duration, minute	1.01	1.00-1.01	0.0730

Bonferroni correction was performed. *p < 0.01, **p < 0.002, ***p < 0.0002. NS, Not significant; ICU, intensive care unit; DNI, delta neutrophil index.
