# Supplementary material for: Association of Delta Neutrophil Index with the 30-day Mortality in Adult Cardiac Surgical Patients
Source: Int J Med Sci. 2024 Jul 1;21(9):1730–7. doi: 10.7150/ijms.97400 (PMC11241090; doi:10.7150/ijms.97400)
Supplement: Supplementary file 1 — Supplementary table. [file ijmsv21p1730s1.pdf]

Table S1. Result of logistic regression analysis by enter method

Variable	Coefficient	95% CI of coefficient	P value
**On admission to the ICU**			
DNI, %	1.06	1.00–1.12	NS
Emergency surgery**	4.81	1.89–12.25	0.0010
History of cardiac surgery	1.51	0.49–4.67	NS
Hb < 10g/dL	2.79	1.15–6.79	NS
Cr > 1.3mg/dL	1.66	0.68–4.03	NS
BNP > 100pg/mL	1.47	0.52–4.16	NS
CPB duration, minute***	1.01	1.01–1.02	< 0.0002
**Postoperative, 12 hrs**			
DNI, %**	1.11	1.05–1.17	0.0002
Emergency surgery*	4.18	1.57–11.12	0.0042
History of cardiac surgery	1.51	0.48–4.76	NS
Hb < 10g/dL	2.89	1.17–7.09	NS
Cr > 1.3mg/dL	1.67	0.66–4.19	NS
BNP > 100pg/mL	1.41	0.48–4.16	NS
CPB duration, minute***	1.01	1.01–1.02	<0.0002
**Postoperative, 24 hrs**			
DNI, %*	1.09	1.03–1.15	0.0024
Emergency surgery*	3.83	1.38–10.60	0.0099
History of cardiac surgery	1.72	0.53–5.57	NS
Hb < 10g/dL	2.58	1.01–6.57	NS
Cr > 1.3mg/dL	1.68	0.65–4.34	NS
BNP > 100pg/mL	1.34	0.45–3.95	NS
CPB duration, minute**	1.01	1.00–1.01	0.0010
**Postoperative, 48 hrs**			
DNI, %***	1.17	1.08–1.27	<0.0002
Emergency surgery*	5.20	1.67–16.26	0.0045
History of cardiac surgery	1.73	0.34–8.71	NS
Hb < 10g/dL	2.44	0.79–7.57	NS
Cr > 1.3mg/dL	1.33	0.43–4.11	NS
BNP > 100pg/mL	1.59	0.41–6.08	NS
CPB duration, minute	1.01	1.00–1.01	NS
**Postoperative, 72 hrs**			
DNI, %**	1.42	1.16–1.73	0.0005
Emergency surgery*	5.52	1.69–17.99	0.0046
History of cardiac surgery	1.03	0.15–6.94	NS
Hb < 10g/dL	1.66	0.50–5.49	NS

Cr > 1.3mg/dL	1.20	0.37–3.90	NS
BNP > 100pg/mL	1.52	0.40–5.79	NS
CPB duration, minute	1.01	1.00–1.01	NS

Bonferroni correction was performed. *p<0.01, **p<0.002, ***p<0.0002. NS, not significant; ICU, intensive care unit; DNI, delta neutrophil index.
